# Chilean public attitudes towards beef production systems

**DOI:** 10.1371/journal.pone.0284080

**Published:** 2023-04-26

**Authors:** Valentina Mansky de la Fuente, Daniel Enriquez-Hidalgo, Dayane Lemos Teixeira, Rafael Larrain, Maria Jose Hötzel

**Affiliations:** 1 Pontificia Universidad Católica de Chile, Facultad de Agronomía e Ingeniería Forestal, Santiago, Chile; 2 Universidade Federal de Santa Catarina, Laboratorio de Etologia Aplicada, Florianópolis, Brazil; 3 Bristol Veterinary School, University of Bristol, Langford, United Kingdom; 4 Rothamsted Research, Sustainable Agriculture Sciences, North Wyke, United Kingdom; 5 Instituto de Ciencias Agroalimentarias, Animales y Ambientales (ICA3), Universidad de O’Higgins, San Fernando, Chile; 6 Department of Animal and Agriculture, Hartpury University, Gloucester, United Kingdom; 7 Center of Applied Ecology and Sustainability (CAPES), Santiago, Chile; Universidade Federal de Mato Grosso do Sul, BRAZIL

## Abstract

Much is discussed about the characteristics, efficiency, and externalities of indoor housing and pasture-based beef production systems, but little is known about how these features influence public attitudes towards beef production. This study aimed to explore Chilean citizens’ attitudes towards beef production systems and their underlying reasons. Citizens (n = 1,084) were recruited to participate in a survey and given information about one beef production system: indoor housing, continuous grazing or regenerative grazing. Participants had more favourable attitudes (from 1 = most negative attitudes to 5 = most positive attitudes) towards pasture-based systems (regenerative grazing = 2.94; continuous grazing = 2.83) than towards indoor housing (1.94), mainly due to concerns with animal welfare and environmental impacts. Productivity was not as important as the other sustainability aspects for participants as they were not willing to do that trade-off. Support for beef production may benefit if production systems adopt characteristics that are perceived by the public as positive for the environment and animal welfare.

## 1. Introduction

Beef production systems differ in characteristics like system productivity and land, animal and housing management practices, entailing different animal welfare and environmental impacts [1, 2]. Due to these differences, the production efficiency of the different systems, understood as the animal productivity obtained using the same amount of input resources, may also differ [3]. Additionally, the efficiency of resources used in a given system may influence productivity and environmental impact per unit of product provided [4, 5].

Three commonly used types of beef production systems globally are indoor housing, continuous grazing and regenerative grazing of which the last two are pasture-based systems. The indoor housing system, also known as feedlot, is considered highly efficient in terms of productivity and use of resources [5], but it might have some negative effects like soil erosion and land degradation, loss of biodiversity, water pollution and usually has low carbon sequestration [6–8]. Pasture-based systems, in contrast, are considered less efficient than the indoor housing system [3]. In continuous grazing systems, characterized by low stocking densities, cattle graze the same pastures for at least a long part of the grazing season (usually weeks to months), with animals changed only occasionally to different fields or paddocks [9]. Regenerative grazing systems use short grazing intervals, long recovery periods and high stocking densities, attained by a frequent change of the cattle among small paddocks. Continuous grazing, the more widely used system, commonly leads to bare soil and soil degradation, biodiversity loss and low carbon sequestration or even emissions, which makes it the most unsustainable system [6], In contrast, regenerative grazing can regenerate soil, increase biodiversity and reach higher rates of soil carbon sequestration with little or no pollution, as well as increase animal productivity compared to continuous grazing [10–14].

The world’s human population growth and associated income per capita increase are pushing a growing demand for animal food products, including beef [15, 16]. In the last decades, beef production has increased, pushed by the growth of intensive indoor housing cattle production systems [17]. As a result, global beef production outcomes regarding environmental, animal welfare, social, and economic aspects have also changed [18–21]. The modern beef production systems have been effective regarding productivity, and with the looming environmental crisis and increasingly prominent concerns towards sustainability, there is a debate whether productivity is the pillar on which the systems must continue to be developed, given that increased productivity may increase other externalities [22].

With the intensification of animal production and increased productivity, citizens have become increasingly concerned about animal welfare and ethical matters and are demanding greater regulation on the topic [23]. Citizens in developing countries have expressed concerns with animal housing and rearing systems [24–26] and these aspects need to be considered in developing a constructive dialogue between industry and consumers.

To our knowledge, citizens’ attitudes towards the different beef productive and housing systems have not been previously investigated. In previous research the most studied topic has been the attitude of the public towards the access of animals to pasture (e.g. [24, 27, 28]). Consumers have more favourable attitudes towards pasture-based beef and are willing to pay higher prices for it [24, 29–32], compared to beef produced in indoor housing systems. However, public attitudes to different beef production systems may be influenced by information of the systems’ characteristics and externalities. Therefore, this study aimed to explore Chilean citizens’ attitudes towards different beef production systems.

## 2. Materials and methods

### 2.1 Survey and data collection

The Research Ethics and Safety Board at the Pontificia Universidad Católica de Chile approved the study and granted a Certificate of Exemption (n. 170906008) due to the type of questions and the anonymity of the participants. This study consisted of a survey applied to 1145 Chilean participants and was carried out in two parts, differing in the type of participants’ recruitment. The first part of the recruitment, which was interrupted by the outbreak of Covid-19, was done face-to-face in February 2020 in Santiago de Chile (n = 281); the second part was done online, between April and May 2020 (n = 864).

Data collection was conducted using a self-administered questionnaire, with no interaction between recruiter and respondent after the acceptance to participate. Face-to-face participants were recruited personally in public spaces with a large influx of people (civil registry offices, bus stations, outside notary offices and the international airport), where people were awaiting or had free time. The online version of the questionnaire was carried out through Google Forms Online platform (www.docs.google.com). Online participants were invited to participate through different social networks such as Whatsapp groups, Instagram, Facebook and by email lists sharing the questionnaire link and inviting participants to respond and share the survey. In all cases participants were invited to complete a survey about animal production, with no specification of the nature of the issue, to reduce self-selection bias. Only participants that were at least 18 years old and had Chilean nationality participated of the study. The questionnaire was in Spanish, translated to English for preparation of this article. The identity of the participants was not required.

The first 15 questionnaires collected were conducted as a pilot study and answers and comments were discussed among the research team, reviewed and some minor refinements were made to the questionnaire. The final questionnaire included 3 closed questions and 1 open-ended question related to the objective of the study, and sociodemographic questions to characterize the participants. Participants who agreed to participate in the survey were asked to read an informed consent that had to be accepted before starting the questionnaire. It clarified the purpose of the investigation, ensured the anonymity of the participation (personal data identifying participants were not collected) and that the information collected was going to be used exclusively for research purposes. It also explained that participants did not run any risk by participating in the investigation, that there was no compensation for doing so and that they could withdraw at any time if they wished, by not handling back the paper questionnaire or sending the online version, without any consequence.

Participants were randomized into three groups, which corresponded to different housing and beef production systems: one group received information about the indoor housing system, a second group about continuous grazing and a third group about regenerative grazing. Participants were first invited to read a description about the beef production system that they were assigned, including information about how animals are housed, how much space they have, and some of the main management practices used in each system. It also included information about each system’s productivity, greenhouse gases emissions per unit of product, water contamination, soil erosion, biodiversity and carbon sequestration. Information provided in each survey was as follows:

**Indoor housing system:** *“The most common beef productive systems in Chile are indoor housing and grazing*, *which can be extensive or intensive*. *Grazing is the system in which cattle is kept in pastures and get their feed directly from it*. *Indoor housing is the system in which cattle is kept together within closed spaces or sheds*. *A smaller space per animal and a smaller area for the production of feed that covers the nutritional requirements of the animals is allocated in comparison to grazing systems*. *This is a highly productive system*, *generally generating low greenhouse gas emissions per kilogram of meat produced*, *but with low soil carbon sequestration*. *This system usually generates a high degree of water contamination and soil erosion*. *Furthermore*, *this system generally reduces the biodiversity in the ecosystem”*.

**Continuous grazing**: *“The most common beef productive systems in Chile are indoor housing and grazing*, *which can be extensive or intensive*. *Grazing is a system in which cattle is kept in pastures and they get their feed directly from it*. *Continuous grazing is a system in which cattle is left free with a large space per animal in the pastures*, *allocating a large area for feed production*. *This system has low productivity*, *generally generating high greenhouse gas emissions per kilogram of meat produced*, *intermediate or low carbon sequestration and intermediate or high contamination and soil erosion*, *and also results in low biodiversity”*

**Regenerative grazing**: *“The most common beef productive systems in Chile are indoor housing and grazing*, *which can be extensive or intensive*. *Grazing is a system in which cattle is kept in pastures and they get their feed directly from it*. *Intensive or regenerative grazing is a way of managing grazing in which a small space is assigned for the animals for a short period before they are moved to the next space*, *controlling the time the animals spend in each paddock*. *Thus*, *feeding area is usually intermediate*, *ensuring that the cattle meet their nutritional requirements*. *This system has an intermediate productivity*, *so it also generates intermediate greenhouse gases emissions per kilogram of meat produced*. *It is characterized by high carbon sequestration*, *low water pollution and low or no soil erosion*, *and it can also lead to increased biodiversity*.*”*

### 2.2 Participants’ attitudes towards beef production systems

Participants were asked their opinion towards the beef production system they had read about. First, they were asked if they approved the housing and beef production system they have just read about, and then they were asked in an open question to justify their answers. The following questions asked if they approved that the beef they commonly consume came from the system described in the text and if they approved that the system should be the beef production system of the future. Possible responses were the same for the three closed questions, in a 5-point-likert scale (1: totally disapprove, 2: disapprove, 3: not approve or disapprove, 4: approve or 5: totally approve).

### 2.3 Participants’ socio-demographics and characterization

The following three questions addressed participants’ socio-demographic information relating to their gender (female; male; other), age (18–25; 26–35; 36–45; 46–55; 56–65; over 66 years old) and education (incomplete school education, complete school education, incomplete technical education, complete technical education, incomplete undergraduate education, complete undergraduate education or postgraduate education). They were also asked about their meat consumption habits (omnivore; vegetarian; vegan; other). Finally, all the respondents were asked if they had any type of relationship with animal production (yes, I grew up in a place related to animal production; yes, I currently have some kind of relationship; or no relationship).

## 3. Statistical analysis

From the initial 1145 questionnaires completed, 61 were excluded for various reasons, including responses from non-Chileans, low representation of ‘other’ gender (18), incomplete surveys and responses that were not readable or understandable, resulting in 1084 usable responses (261 face-to-face and 823 online).

Responses to the face-to-face questionnaire were transferred to the platform Google Forms Online and all information was automatically transcribed to a Microsoft Excel (version 2013) sheet. Descriptive statistics for the responses were calculated using Microsoft® Excel for Mac and all other statistical analyses were conducted using SAS 9.3. For the purpose of the statistical analysis, participants were classified as with university education (complete or on-going) or no university education; as meat consumers (if they consumed beef, pork, poultry, or small ruminants) or not meat consumers; and if they had some kind of relationship with animal production or not. Age 56–65 and over 66 years old, as well as professional involvement and grew up in an agriculture environment were respectively grouped, due to the low number of participants in these categories.

An initial exploration for the three closed questions regarding participants’ approval of the systems was done using Spearman Correlation coefficients. As the responses were highly correlated (R^2^ > 0.82; P < 0.001), they were averaged to create an “attitude” construct ranging from 1 (most negative attitude) to 5 (more positive or favourable attitude). The attitude construct was normally distributed as evaluated using the Univariate procedure. A generalized linear model (GLM) was then used to evaluate the effects on the attitude construct, including beef production system (indoor housing, continuous grazing and regenerative grazing), participant’s gender, age, questionnaire type (face-to-face or online), educational level, meat consumption and involvement with animal production as explanatory variables in the model.

### 3.1 Thematic analysis

The analysis of the open-ended question was submitted to coding reliability thematic analysis [33]. To ensure that the coding of themes was appropriate, initially three readers (VMdlF and two other independent individuals) analysed 50 random responses for each of the three treatments and independently developed codes. These responses were read for familiarization with the data; codes were generated inductively and conceptualized into themes by the three coders, who then shared and compared their results and discussed discrepancies until agreement was reached and titles and definitions for each theme were generated. Then, VMdlF coded all the open answers. Some examples of answers given by the participants for each of the themes are presented in the results. They are presented using codes according to the system they were asked about (IH: Indoor housing; CG: Continuous grazing; RG: Regenerative grazing) together with the participation number in order to be able to identify the answers if necessary (e.g., IH1053: indoor housing answer from participant number 1503).

## 4. Results

Socio-demographic data are shown separately for the face-to-face and the online parts of the questionnaire (Table 1). Most participants were meat consumers, not involved with animal production, and had on-going or completed university education.

**Table 1:** Socio demographic information of survey participants for the face-to-face (n = 261) and the online version of the questionnaire (n = 823).

	Face-to-face		Online		Total	
Variable	n	(%)	n	(%)	n	(%)
Gender						
Male	109	42	285	35	394	36
Female	152	58	538	65	690	64
Age						
18 to 25 years old	78	30	275	33	353	33
26 to 35 years old	73	28	185	23	258	24
36 to 45 years old	48	18	115	14	163	15
46 to 55 years old	32	12	135	16	167	15
56 years old and over	30	12	113	14	143	13
Beef production system information						
Indoor housing	100	38	252	31	352	33
Continuous grazing	96	37	299	36	395	36
Regenerative grazing	65	25	272	33	337	31
Meat consumption						
Yes	226	87	640	78	866	80
No	35	13	183	22	218	20
Involvement with animal production						
No	227	87	720	87	947	87
Yes	34	13	103	13	137	13
Education						
No university education	78	30	134	16	212	20
University education complete or on-going	183	70	689	84	872	80

### 4.1 Attitudes towards beef production systems

Attitude was more negative (lower value) towards the indoor housing system than to continuous grazing, which was lower than for regenerative grazing (1.94 vs. 2.83 vs. 2.94, respectively, SEM 0.078; P < 0.001). In general, despite the overall low values observed for the attitude construct, participants that were involved in animal production had a more favourable attitude towards all beef production systems than those without involvement (2.76 vs. 2.38, SEM 0.068; P < 0.001). There were interactions between gender and meat consumption of participants (Table 2): males had a more positive attitude to indoor housing than females (P < 0.01), but both had more positive attitude to both pasture-based systems than to indoor housing (P < 0.001). While meat consumers had more positive attitude towards the regenerative grazing than the continuous grazing (P < 0.01), participants who did not eat meat had similar attitudes towards both pasture-based systems. Questionnaire type (online or face-to-face), education level and involvement with animal production had no effect on participants’ attitude toward the beef production systems (P > 0.05).

**Table 2:** Participants’ attitude towards the different beef production systems (n = 1084).

	Beef production system				P-value	
	Indoor housing	Continuous grazing	Regenerative grazing	SEM	Main effect	Interaction with beef production systems
Gender					< 0.05	< 0.05
Male	2.13 (12)1	2.84 (12)	2.96 (12)	0.107		
Female	1.76 (20)	2.82 (14)	2.92 (19)	0.085		
Meat consumption					< 0.001	< 0.001
Yes	2.36 (26)	3.41 (28)	3.74 (24)	0.070		
No	1.53 (7)	2.24 (8)	2.13 (7)	0.126		

SEM = Standard error of the mean.

Attitude construct = From 1 to 5 (1 = Totally disapprove; 2 = Disapprove; 3 = I do not approve or disapprove; 4 = Approve; 5 = Totally approve).

^1^Percentage of participants for each category in parenthesis.

Nine themes were identified as justifications for the citizens’ attitudes towards the production systems (Table 3). Some responses were assigned into multiple themes and some responses had no valid justification, so they were classified as “no justification” and were excluded from the analysis (n = 1187). The two main themes covered by participants for approving or disapproving their respective beef production system were animal welfare (25%) and impacts on the environment (18%). Productivity was the most mentioned theme by participants approving of the indoor housing (87%), animal welfare by those approving of the continuous grazing (65%) and the environment the most mentioned by participants approving of the regenerative grazing system (51%). Animal welfare related aspects were the most mentioned reasons for disapproval of the indoor housing (43%) and regenerative grazing systems (38%), while reasons related to the environment were the most commented for the disapproval of the extensive grazing system (60%).

**Table 3:** Emerging themes in participants’ justification for approval or disapproval the different beef production systems and percentages of mentions.

	Indoor housing			Continuous grazing			Regenerative grazing			
	Disapp	Not app or disapp	App	Disapp	Not app or disapp	App	Disapp	Not app or disapp	App	Total mentions for each theme
**Animal welfare**	12.6	0.2	0.3	1.3	0.3	9.9	4.4	0.4	4.6	**34.0**
*(Space*, *freedom to move*, *adequate feeding*,										
*animals’ feelings*, *stress*, *shelter*, *health and the treatment that humans give them)*										
**Environment**	5.7	0.0	0.0	9.4	0.1	1.5	1.7	0.2	7.5	**26.1**
*(Contamination*, *greenhouse gases*, *biodiversity*, *soil erosion*, *water pollution*, *air pollution and odours)*										
**Ethical issues**	4.1	0.0	0.0	1.9	0.0	0.6	1.9	0.1	0.1	**8.7**
*(Respect*, *animal rights*, *moral and ethics)*										
**Disagreement with meat production**	1.6	0.0	0.0	2.6	0.1	0.0	2.7	0.0	0.0	**7.0**
*(No meat should be produced)*										
**Productivity**	0.5	0.3	1.9	1.2	0.0	0.5	0.1	0.2	1.6	**6.3**
*(Efficiency*, *sustainability and profitability)*										
**Natural system**	1.6	0.1	0.0	0.1	0.0	1.7	0.4	0.1	0.8	**4.8**
*(Natural feeding or housing)*										
**Lack of knowledge**	0.1	0.7	0.0	0.0	2.0	0.0	0.1	1.1	0.3	**4.3**
**Indifference**	0.0	1.2	0.0	0.0	1.1	0.1	0.0	1.0	0.0	**3.4**
**Beef quality**	1.4	0.1	0.0	0.2	0.0	0.7	0.1	0.1	0.3	**2.9**
*(Nutritional and organoleptic quality)*										
**Other**	0.5	0.1	0.2	0.1	0.0	0.2	0.3	0.0	1.1	**2.5**
**Total**	**28.1**	**2.7**	**2.4**	**16.8**	**3.6**	**15.2**	**11.7**	**3.2**	**16.3**	**100.0**

Attitude construct = From 1 to 5 (Disapp = Totally disapprove (0) and disapprove (1); Not app or disapp = I do not approve or disapprove (2); App = Approve (3) and Totally approve (4)).

Answers without any justification were not included in this analysis.

### 4.2 Animal welfare

Space and freedom to move were the most common issues raised by participants [e.g., *Due to the conditions in which the animals are*, *without fresh air or space to run* (IH1053, response = totally disapprove); *For me*, *the best thing would be that animals lived in a free space where they can freely eat what they want*, *without rules* (RG612, response = totally disapprove); *Free*, *happy cows* (CG89, response = totally approve)]. In general, participants associated lack of space and freedom to move with animal suffering and stress or vice versa [e.g., *Because the animal is not stressed*, *it is free in a wide space* (CG357, response = approve)]. For 30% of participants that approved regenerative grazing animal welfare was a concern [e.g., *Animals subjected to less stress conditions*, *are free grazing with feeding and drinking conditions according to requirements* (RG304, response = totally approve)], whereas 38% disapproved it for the same reason [e.g., *Animals need more space to live* (RG63, response = disapprove); *Little space for each animal* (RG103, response = totally Disapprove)].

### 4.3 Environmental impact

The second most common theme to justify the level of approval of the different systems was the impact of the beef production system on the environment (18% of responses), with participants addressing different topics such as pollution, greenhouse gases, and soil erosion. Environmental impact was the most common reason for the disapproval of continuous grazing and a strongly negative attitude was observed towards the impact that this system generates on the environment [e.g., *For the damage they cause to the environment where they graze*. *Livestock eat everything green (including perhaps native species) and in this way erode the soil and damage the ecosystem* (CG125, response = disapprove); *Because food production should not be a reason to damage the environment*, *since originally nothing was like that* (CG411, response = disapprove)]. Some participants associated the issue of environmental impact with productivity [e.g., *It implies emission of polluting gases and low productivity*. *You could have better productivity and less contamination with another system* (CG646, response = Totally disapprove)]. The environmental impact of the regenerative grazing was the main reason for participants’ approval of this system [e.g., *Less greenhouse gases emissions*, *protects the soil and water allowing a rational use of the meat resource as protein consumption* (RG131, response = approve); *According to the explanation*, *this system is more sustainable*. *The reduction of greenhouse gases is important*, *I have read about this factor*, *and it is relevant how the animal explosion is increasing considering that most of the greenhouse gases come from this productive sector* (RG953, response = approve)].

### 4.4 Productivity

The third most frequent theme was productivity, mentioned by 87% of the participants that approved the indoor housing system [e.g., *It has negative but also positive impacts*. *It is important to increase the productivity of production systems*, *like this system does* (IH58, response = approve); *The higher the production*, *the lower the costs for the population* (IH81, response = approve)].

### 4.5 Ethical issues

Ethical justifications were mainly used by the participants that disapproved the production systems they were analysing [e.g., *For ethical reasons*, *I think the abuse of animals is very serious* (IH823, response = totally disapprove)]. Responses mentioning animal rights were also classified in this theme [e.g., *From the point of view of animal rights*, *I do not agree with indoor housed as a mean of production*, *although I am not a vegetarian*, *I do not endorse the idea of animal production in quantity only for profit without taking into account what an animal means*, *even more the stress to which they are subjected*, *which then affects the people who consume it…* (IH46, response = totally disapprove); *Animals are living beings that have the same right as human beings to be or feel free* (RG396, response = totally disapprove)].

### 4.6 Beef quality

The quality of the final product from the different systems theme was only mentioned in 2.9% the responses. Some participants disapproved of these systems due to the perceived quality of the final beef [e.g., *I imagine that the beef is better if the cattle graze freely* (IH214, response = disapprove); *For fattening cattle it is not necessary to have a large field*, *the less the exercise the better the quality of the meat* (CG139, response = totally disapprove) and some participants approved the grazing systems for the quality of the beef [e.g., *The truth is that thinking only about the business and the quality of the beef*, *I think that this system is more profitable and produces a softer beef* (RG221, response = totally approve)]. Nonetheless, no participant approved the indoor housing product for the final quality of the product.

### 4.7 Naturalness

The extensive grazing system was perceived as the most natural and which received the most approvals for this reason [e.g., *The animal can develop naturally* (CG880, response = approve); *It is the natural way that animals must develop in the ecosystem naturally* (CG34, response = approve)], but some comments were similar for the regenerative grazing [e.g., *Animals should relate naturally to their environment*., *like in this system* (RG611, response = approve). The indoor housing, in contrast, was the system perceived as the least natural [e.g., *It is not the natural cycle of production that is needed*, *I feel that it is done in this way to satisfy an exaggerated demand*, *with respect to the real consumption needs (if there are any)* (IH258, response = totally disapprove)].

### 4.8 Indifference and lack of knowledge

Some participants expressed their indifference to justify that they will eat the beef regardless of the system in which it is produced [e.g., *I like meat*, *I don’t care how they produce it* (CG857, response = not approve or disapprove); *It keeps me indifferent because I am a meat consumer regardless of its productive process* (RG1505, response = not approve or disapprove)]. Other participants were indifferent because they considered that it is not their concern how the animals are raised and/or the meat is produced [e.g., *I eat meat*, *I don’t raise cattle* (IH561, response = not approve or disapprove); *I am indifferent because it does not concern me* (RG603, response = not approve or disapprove)]. A total of 47 participants said that they were unaware of the subject to justify why they were indifferent to the issue [e.g., *I am not informed on the subject* (RG287, response = not approve or disapprove); *I do not have more information to approve or reject this type of grazing* (CG493, response = not approve or disapprove); *I am unaware of the subject to take a clear position*, *I am not indifferent but I do not have the necessary clarity* (IH1032, response = not approve or disapprove)]

### 4.9 Disagreement with meat production

Some participants disapproved beef production regardless of the system used [e.g., *I disagree because I start from the assumption that we should not consume animals* (IH335, response = totally disapprove*); I am against food based on killing the life of an animal in a violent way*, *in addition*, *that the livestock industry is the one that pollutes the most* (CG687, response = totally disapprove); *Even if they keep them (animals) in open places where they graze freely*, *it is still for them to kill them afterwards*. *What’s the point of keeping you in a nice place if they are going to kill you anyway*? (RG104 response = totally disapprove)]

## 5. Discussion

Participants had less negative attitudes towards the pasture-based beef production systems compared to the indoor housing system, similar to other surveys with citizens from developed and developing countries [29, 34, 35]. Animal welfare and environmental impacts were the main reasons given by participants to either support, reject or oppose the three systems. Both issues were leading reasons for the greatest positive attitudes towards continuous and regenerative grazing, as well as for the negative attitudes towards indoor housing. However, the environmental impact of the continuous grazing system also generated the highest negative attitudes towards this system. Only few people disapproved the pasture-based systems based on concerns with productivity, which was also the most frequent reason for approval of indoor housing. Increased productivity has environmental and animal welfare pros and cons [18, 19, 36] so we assume that participants were not eager to accept a trade-off between animal welfare and environmental issues. The results from our study highlight a conflict between the beef industry’s approach of intensification to increase productivity and the ethical aspects of the production systems considered important for the lay citizens.

Animal welfare is one of the most important quality attributes in beef for people [37], and it is also one of the main reasons underlying preferences for pasture-based systems [34]. Therefore, it is not a surprise that it was a topic of great concern for the participants. Our findings reiterate that animal welfare is a significant aspect in food animal production for consumers, which needs to be considered in order to ensure the long-term sustainability of the industry [6].

Citizens often consider space per animal, freedom of movement, grazing and access to pasture amongst the most important animal welfare attributes [27, 28], and animals in systems with those attributes are seen as having a more natural life [28]. Those attributes were mentioned by participants as justification to approve the grazing systems and also to disapprove the indoor housing and the regenerative grazing, suggesting that participants believed that the later system limits animal freedom. Indeed, the greater space availability for cattle in continuous grazing than in the other systems was the most cited reason for the positive attitude towards animal welfare in that system. In addition, the lack of knowledge about beef production systems and low level of involvement with animal production of the participants may have resulted in a misinterpretation of the space availability or stocking density in the systems, since the perception of space is not easily communicated and bias can be generated between the space imagined and real space availability [38, 39]. Studies with images or visits to the different beef production systems (i.e. [40–42]) may have different impact on citizens’ attitudes, which warrants further investigation.

Although participants perceived beef cattle welfare as better in pasture-based than in indoor housing systems, some issues associated to animal welfare require a more careful analysis. On the one hand, pasture-based systems may reduce the risk factors for some cattle health problems, such as acidosis, liver abscesses and hoof related pathologies [43], may allow animals to express their natural behaviours, such as grazing, better lying/resting behaviour, and result in calmer animals [44–46]. On the other hand, cattle in pasture-based systems may be under greater risk of being exposed to parasites, extreme weather, and experience malnutrition [44], especially under overstocking situations [45]. Nevertheless, possible issues that could reduce animal welfare in the pasture-based systems were not mentioned in the evaluation of the systems, given that citizens might not be aware of them [47] and most of our participants were not related to animal production. Moreover, to the best of our knowledge, there are no studies comparing beef cattle welfare in continuous grazing and regenerative grazing systems. Such information is needed to support an evidence-based discussion of the issue.

Participants were more aware of the environmental impact of meat production compared to previous studies [48, 49]. The environmental awareness of participants concerning beef production identified in our results could be related to the increasing media coverage of the impact of food and meat production on the environment during the last years, based on reports released about the effect of beef production [50, 51] and the fact that a reduction in meat consumption is repeatedly proposed as a way to mitigate greenhouse gases emissions of anthropogenic origin [52, 53]. These proposals, however, ignore the fact that some production systems, instead of aggravating the environmental problems, can positively contribute to mitigate the impacts of food and meat production, given that some beef production systems such as regenerative grazing can have positive consequences in the ecosystem such as land restoration, improved resources cycles and biodiversity, and soil carbon sequestration, helping to mitigate climate change [8, 10, 54]. The positive association between the perception of environmental benefits of the systems and attitudes towards beef production systems suggests that environmental impact of the food production systems is a relevant concern for society and may be a reason for support of regenerative grazing systems [55, 56].

In order to meet the increasing demand for animal food products [15, 16], producing more and increasing efficiency has been seen as one of the main solutions to supply enough food, as proposed by the concept of “sustainable intensification” [5]. However, sustainable intensification also has its environmental pros and cons [19, 36]. Participants did not seem to accept a trade-off between the higher productivity and the environmental cost of the greenhouse gas emissions generated and the other environmental consequences, as participants expressed more favourable attitudes towards pasture-based systems, described in the information provided as less productive than indoor housing systems and that their low productivity was not a main reason for their disapproval. This suggests that the participants preferred more sustainable production over productivity. It would be interesting to study if consumers would be willing to pay more for a product coming from these systems or to reduce their level of consumption, as has been suggested by others [57].

### 5.1 Issues related to participants’ recruitment and influence of demographics on responses

The convenience sample used in this survey does not represent the Chilean population, as participants were arguably younger and more educated than the average of the national country population. Through this recruiting, though, we were able to reach citizens from different places, which allowed us to investigate associations between attitudes and sociodemographic characteristics. Thus, even if the results cannot be generalized to the general population of Chile, they contribute novel understanding of Chilean citizens’ knowledge, preferences, and perceptions regarding beef production systems.

Online recruitment is believed to create a bias, as it only reaches people with Internet access [58]. Additionally, our sample of participants contained of a high proportion of females and young people with university education, similar to samples obtained in previous surveys carried out online on the same subject [59–61]. However, responses were not influenced by type of recruitment, even though the online portion of the sample had a much higher proportion of females, younger people and participants with complete or on-going university education compared to the face-to-face sample. This may be due to the fact that, despite these limitations, our sample contained a good diversity. This diversity and the number of participants allowed us to analyse the influence of some demographic aspects on the outcome variables. Females had more negative attitudes towards all beef production systems, as well as more negative perceptions towards beef consumption than males. These results are in agreement with other studies that have reported that women have more positive attitudes towards farm animal welfare, and more often follow low meat and meatless diets, vegetarianism and ethical food choices (e.g., [62–64]). Participants involved in animal production, in contrast, had more favourable attitudes towards the three beef production systems, in agreement with other studies that showed the different values regarding animal production and husbandry that farmers and lay urban citizens have [65–67], which may be a consequence of urbane lifestyle and low awareness of animal production systems [68, 69]. Participants who identified as vegetarian or vegan expressed a more negative attitude towards the three systems than meat consumers. Vegetarians have more negative attitudes towards the production and consumption of meat than meat eaters, and vegans have even more negative attitudes than vegetarians [64, 70], as their beliefs about meat consumption is reflected in their meat-avoidance behaviour [71].

## 6. Conclusion

Participants had more favourable attitudes towards the pasture-based systems than to the indoor housing system, where cattle have no access to pasture. The two main reasons underlying the attitudes towards the systems were animal welfare and the environmental impact generated by the beef production systems. For the two previous reasons, participants expressed different attitudes towards the two grazing systems, being more positive to regenerative grazing than to extensive grazing. This means that the public believes animals should have access to pasture and be able to graze, but they are also concerned with the externalities of each type of management. Support for beef production may benefit from adoption of housing and management practices that are perceived by the public as positive for animal welfare and the environment.
